# A Comprehensive Review of School-Based Lifestyle Interventions to Reduce Childhood Obesity in the Eastern Mediterranean Region

**DOI:** 10.7759/cureus.64305

**Published:** 2024-07-11

**Authors:** Ihdaa J Abdulwahab, Noha A Alzahrani, Jumana H Khouja

**Affiliations:** 1 Preventive Medicine Department, Saudi Board of Preventive Medicine, King Abdulaziz Medical City Jeddah, Jeddah, SAU; 2 Preventive Medicine, King Abdullah International Medical Research Center, Jeddah, SAU

**Keywords:** eastern mediterranean region, lifestyle interventions, school-based interventions, children, obesity

## Abstract

Childhood obesity prevalence has increased worldwide and substantially in the 22 countries of the Eastern Mediterranean Region (EMRO). Weight-related interventions are urgently required in these countries to tackle childhood obesity and its related consequences. There has been no review to date of obese children in the Eastern Mediterranean Region. This review discusses the different school-based lifestyle interventions conducted among obese children in the EMRO and assesses the applicability of future programs in Saudi Arabia. A thorough search of the literature was conducted on PubMed. A total of 170 studies were found, and eight of them were included in this review. The included studies were all randomized controlled trials or quasi-experimental. This review article showed that school-based lifestyle interventions may reduce childhood obesity by integrating interactive learning about healthy diet and physical activity within a whole school approach, involving children and their parents, modifying the school environment, and facilitating a workshop on healthy food preparation. To motivate children to change their behavior, it is crucial to meet with parents in person and utilize technology and rewards. School-based lifestyle programs can lower childhood obesity by involving all relevant parties, such as families and schools, and using reliable instruments to track results to establish a healthy community. In order to confirm these findings, more research is required for a longer period of time, more than six months.

## Introduction and background

Obesity among children is considered one of the greatest health problems facing the world today. Obesity among children is considered one of the greatest health problems facing the world today. According to the World Health Organization, children between the ages of five and 19 are considered overweight if their body mass index (BMI) for age is greater than one standard deviation (SD) or obese if their BMI for age is greater than two SD [1], while the Centers for Disease Control and Prevention (CDC) defines overweight among children aged two to 19 as lying between the 85th and 95th percentiles and obesity as equal to or greater than the 95th percentile [2]. The WHO reported in 2022 that globally, 8.24% of children and adolescents aged between five and 19 are obese, compared to 11.9% in the Eastern Mediterranean Region (EMRO) [3]. In 2019, the National School-Based Screening Program (NSBSP) in Saudi Arabia reported that the prevalence of overweight and obesity among primary school students was 10.5% [4]. Recently, a national health survey in 2023 reported that nearly 18% of children and adolescents (<15 years) were overweight or obese [5].

Childhood obesity can lead to adult obesity [2]. Further, it's a leading cause of noncommunicable diseases such as hypertension, diabetes mellitus, dyslipidemia, metabolic syndrome, cardiovascular, musculoskeletal, and pulmonary complications [6-8]. As a consequence, the NSBSP program has been implemented in schools since 2017 in Saudi Arabia, targeting all levels of education (primary, intermediate, and secondary). However, there are many factors contributing to the rise in obesity among children, including genetics, lifestyle, environment, socio-economic status, schools, education, and behavioral factors [9]. Reducing calorie intake and increasing physical activity through lifestyle and behavior modification are recommended treatments to promote a healthy BMI [10]. Parental involvement in the treatment of obesity is also crucial for improving children's self-efficacy in the promotion of a healthy diet [10,11].

To our knowledge, no review has been conducted among obese EMRO children ranging from five to 12 years of age. This review summarizes the different school-based lifestyle interventions conducted among obese children and assesses the applicability of future programs to be implemented in Saudi Arabia. Since then, no clinical trials have been done on children aged between five and 12 years of age. 

Methods

Search Strategy

The literature search on PubMed was carried out using the following mesh terms: Child, Student Health Services, School Health Services, school based intervention, prevention and control, Mass Screening, Exercise, Sedentary Behavior, Diet, Diet Food and Nutrition, Pediatric Obesity, Overweight, Body Mass Index, Middle East, Afghanistan, Pakistan, Bahrain, Djibouti, Egypt, Iraq, Jordan, Kuwait, Lebanon, Libya, Morocco, Arabs, Oman, Qatar, Saudi Arabia, Somalia, Sudan, Syria, Tunisia, United Arab Emirates, and Yemen. Search results were filtered based on title, abstract, full-text availability, English studies only, children's age group, and last 10 years of publication between 2014 and March 2024. In our search, we found 170 articles. Of the included studies, eight met our inclusion criteria and were conducted in eastern Mediterranean countries. All studies were randomized controlled trials or quasi-experiments.

Inclusion and Exclusion Criteria

All potential studies were evaluated for eligibility if they met the following criteria: 1) school-based interventions conducted in EMRO countries; 2) targeted school-aged children (five to 12 years); 3) addressed at least one aspect of weight-related lifestyle (physical activity, sedentary behavior, or diet); and 4) included at least one anthropometric measure of body weight as a primary or secondary outcome. The studies were excluded if they did not take place in any of the Middle Eastern countries/the EMRO region, were not intervention studies and done on obese adults.

## Review

This is the first review article examining weight-related interventions at school among children five to 12 years old in Middle Eastern countries. Although Saudi Arabia's high obesity prevalence rate compares to other Middle Eastern countries, no studies have been conducted in the kingdom among children in the same age groups. Therefore, conducting such studies is crucial to implementing an effective, feasible, and applicable early childhood program, since it contributes to primary prevention. Table 1 shows study characteristics such as design, duration, and participants of the included studies in terms of age, gender, sample size, and school. As illustrated in Table 1, two studies were conducted in Iran [12,13], two in Kuwait [14,15], two in Qatar [9,16], and one each in Israel [17] and Lebanon [18]. Out of the eight reviewed studies, five were randomized controlled trials [9,12,14,15,17], two were quasi-experimental [13,16], and one had a mixed-methods design [18]. The study duration ranged from three months to 24 months. For all the included studies, the participants were between the ages of five and 12, except for one study that extended to the age of 14 [15]. The sample size varied from 82 to 374 students. Among the included studies, all students were in primary school [9,12-16,18], except for one where children were referred by a community pediatrician and advertised. All the studies included both boys and girls. The number of schools participating in the studies differed from one [16], six [14], eight [13,18], and 12 [12] schools; two studies did not report the number [9,15], and one study was community-based, not school-based [17]. The interventions were either only conducted at public schools or at both public and private schools, and five studies did not report the school type.

**Table 1:** Characteristics and participants of included studies (n= 8) Abbreviations: RCT = Randomized controlled trial, EG = experimental group, CG = control group, NR = not reported

Author, year, country	Study characteristics and participants
Fernandez-Luque (2017) [9] Qatar	Design & duration: RCT, 6 months
	Age & Gender: 9–12 years, boys & girls
	Sample size: n = 277, EG = 108, CG = 119
	School: several
Amini (2016) [12] Tehran	Design & duration: Cluster RCT, 4.5 months
	Age & Gender: 10-12 years, boys & girls
	Sample size: n = 334, EG = 167, CG =167
	School/s: 12 schools
Khatami (2021) [13] Iran	Design & duration: quasi-experimental, 6 months
	Age & Gender: 6-12 years, boys & girls
	Sample size: n = 180, EG = 90, CG = 90
	School: 8 schools
Allafi (2020) [14] Kuwait	Design & duration: RCT, NR
	Age & Gender: 9-11 years, boys & girls
	Sample size: n = 225, 110 boys & 115 girls
	School: 6 public primary schools
Boodai (2014) [15] Kuwait	Design & duration: Assessor-blinded RCT, 6 months
	Age & Gender: 10-14 years, boys & girls
	Sample size: n = 82, EG = 42, CG = 42
	School: NR
Choudhury S (2018) [16] Qatar	Design & duration: quasi-experimental, 5 months
	Age & Gender: 7-12 years, boys & girls
	Sample size: n = 355
	School: one school
Yackobovitch-Gavan (2018) [17] Israel	Design & duration: open-label RCT, 24 months
	Age & Gender: 5-11 years, boys & girls
	Sample size: n = 270, EG1 = 90, EG2 = 90, CG = 90
	Referral from community pediatrics and advertisement
Habib-Mourad (2014) [18] Lebanon	Design & duration: Mixed (RCT & focus group), 3 months
	Age & Gender: 9-11 years, boys & girls
	Sample size: n = 374, EG = 193, CG = 181
	School: 4 private, 4 public schools

Intervention aspects

Table 2 illustrates the interventions and main findings of the studies. Students in all included studies were taught various health-oriented topics related to nutrition, physical activity, and sedentary behaviors. Some of the studies included parents as well, which had a more significant impact on the outcome, specifically BMI changes. Educational materials are delivered differently, by a health advisor, physician, psychologist, or dietitian. A variety of activities and programs were held during the sessions, including lectures, interactive fun activities, and integration into the school curriculum. 

**Table 2:** Interventions and main findings of the studies. Abbreviations: BMI = body mass index, BAZ = BMI-for-age z-score, BMI-SDS = BMI-standard deviation score, WC = waist circumference, HC = hip circumference, NC = neck circumference, MM = muscle mass, FFM = fat-free mass, FM = fat mass, TSF = triceps skin folds, EG = experimental group, CG = control group, NS = non-significant, PA = physical activity, SCT = social cognitive theory

Reference study	Intervention component	Main outcome
Fernandez-Luque (2017) [9] Qatar	The health coaching and education program had three phases:	BMI: the BMI of children whose moms participated in phases 1 and 2 decreased by 0.058 in EG versus 0.0455 in CG (p = 0.5709).
	1- Health camp:	
	-The weight management camp lasted two weeks and featured nutritional and lifestyle counseling as well as physical and social activities.	
	-For ten days, the students were tasked with photographing their food trays before and after breakfast and lunch.	
	2-Weekend Clubs:	Physical Activity: Children who recorded more active days on actigraphy had a larger drop in BMI (r = -0.263, p = 0.101). Active day is defined as active minutes that are non-zero.
	-Students were reinforced for healthy conduct for ten weeks, four hours each weekend.	
	-An actigraphy sensor was utilized to monitor physical activity and sleep habits. EG=51, CG=16	
	-50 parents were given a cell phone with a pre-configured Instagram account to capture photos with. Instagram tracks social media food photographs.	
	3- Summer Break:	Social media usage: Using Instagram more actively associated with children's BMI (r = -0.179, p = 0.425).
	-For three months, a group of mothers received a mobile intervention via WhatsApp to encourage healthy eating behavior	
	-Face-to-face sessions were scheduled to educate parents about health.	
Amini (2016) [12] Tehran	Education	Anthropometrics: decrease the BAZ (p = .003) and HC (p < .001) in EG. WC increase in both EG & CG with a higher rate among CG (p = .001). NS decrease in TSF for EG (p = .51) but increase in CG (p < .001)
	*Nutrition*: EG: Students received 12 educational sessions (15-45 min. each) once/week about nutrition by a health instructor. CG: no nutrition	
	education EG: parents receive 4 sessions (90 min. each). Once/month about multiple behavioural and lifestyle-related topics. CG: Parents receive a summary of all EG topics in one session	Diet: Significant increase in energy, fat and carbohydrate intake among the EG in comparison to CG
	*Physical activity* Sport sessions last for two hours per week, starting with warm-up and street stretching, then aerobic/resistance exercises. The session ends with rest and a cooldown	Physical activity: EG increased vigorous PA (p < .001), CG increase moderate PA only (p < .001)
	*Other: *Change the menus of schools' canteens to remove high-calorie items such as chocolate, sweet drinks, and ice cream. Replace unhealthy cooking options with healthy alternatives and make healthy food appealing to children	Sedentary behaviours: NS difference in TV time spent (P = 0.08) between groups. NS decrease in computer time spent for EG (p = .1) and moreover increase among CG (P = 0.004).
Khatami (2021) [13] Iran	Educational program It consists of two parts:	Anthropometric measurements:
	1) The 5-2-1-0 healthy habit (HH) involves consuming 5 units or more of fruits and vegetables daily, using electronic devices for no more than 2 hours, engaging in 1 hour or more of physical exercise daily, and avoiding sugary beverages.	-After 6 months, EG and CG showed an increase in BMI (p = 0.024) and weight for age percentile (p = 0.044).
	2) Healthy Eating Plate (HEP):	-The CG showed a significant increase as compared to the EG.
	a) Eat half of your plate of fruits and vegetables.	
	b) Fill a quarter of your plate with whole grains and the rest with protein sources	
	c) Consume a balanced amount of vegetable oils.	
	d) Drink water, tea, or coffee.	
	e) Exercise regularly.	
	- Parents were educated online about weight loss and risk factors for obesity, as well as how to reduce weight and benefit from HH and HEP patterns.	-Girls in EG exhibited greater BMI differences at the end of the research (p = 0.006).
	-A website created to be the source of information for parents. Which include multimedia files, images, and web pages. The materials were uploaded twice a week.	-Boys' BMIs did not differ significantly between the two groups (p = 0.507).
	- After educational material was uploaded, parents were questioned via virtual groups and text messages.	
	- Once a month, phone calls were conducted to address any questions or issues regarding the plan or website.	
Allafi (2020) [14] Kuwait	Physical activity: The researcher used a well-validated digital pedometer (Yamax Digiwalker SW-200, Tokyo, Japan) to measure physical activity by counting steps. Children receive a pedometer at the beginning of sessions. Five sessions of PA each lasting 50 minutes.	Anthropometrics: NS difference in average BMI (p = 0.15) or step count (p = 0.16) between boys and girls
	- Children were divided randomly into 3 groups: Feedback group (FB) only received information about pedometer function; feedback with rewards (FD+R) received information about pedometer function and had an incentive of ten stickers for reaching 3000 steps. While CG participants were not informed about the pedometer's function. Before the session starts, the pedometer is set to zero, and the child is instructed to wear it till the session ends. The pedometer for the control group only was sealed.	Physical activity: The average step count among three groups was: CG (2091±483), FB (2655±577), and FB+R (3429±458). There was a significant difference in the average step count between the FB+R group and both other groups, CG and FB (P < 0.00).
Boodai (2014) [15] Kuwait	Educational program:	Anthropometrics: NS difference between EG and CG regarding BAZ (p = 0.6), WC (p = 0.3), systolic (p = 0.9) or diastolic blood pressure (p = 0.2)
	The intervention was adopted from the Scottish Childhood Obesity Treatment Trial (SCOTT), called "good practice". A physician and nutritionist deliver the program to the students and their parents. Group discussion consists of 6 sessions of 1 hour duration over a 6-month period.	
	The program includes two components:	
	1) Change the behaviours that contribute to children's obesity such as decreasing sedentary habits, using a "traffic light diet" and increasing physical activity.	
	2) Identify the pros and cons of each of the three behavioural modifications (diet, physical activity, and sedentary behavior), Increase motivation to change by recording screen time, walks, and sports in diaries. Also, address the barriers against behavioural change and set a goal regarding change to prevent the relapse	
Choudhury S (2018) [16] Qatar	School-based nutrition initiative:	There were no significant changes observed in the prevalence of obesity and overweight, as well as BMI z scores
	-Face-to-face consultations with school nurses and catering staff to give students feedback on their food selections.	Dietary habits: According to a self-reported questionnaire after the intervention, the following results were observed:
	-Redesigned the school cafeteria with colorful booklets informing students about the benefits of macronutrients such as fruits and vegetables.	-Decrease in rice consumption (P = 0.011)
		-Change in frequency of energy drink intake (P = 0.05)
	-Students have access to cards with information about fruits and vegetables, as well as healthy meal recipes.	-Fruit intake increased by 6% and vegetable intake by 3.5% (P > 0.05).
	-A rewards system in which they receive stamps for choosing healthy foods and a badge for collecting a certain amount of stamps.	-Decrease in unhealthy food intake such as cakes, muffins, and biscuits (P > 0.05)
		Anthropometric measurements: Significant increase in FM by 0.6kg (p = .003), HC by 1.5cm (P < 0.001), NC by 1.2cm (P < 0.001), MM by 1.1 kg (P < 0.001), and FFM by 1.2 kg (P < 0.001).
Yackobovitch-Gavan (2018) [17] Israel	Educational Program:	BMI-SDS:
	-The study had three arms: EG1 (patents only), EG2 (parents-child), and CG.	-After 3 months of intervention, BMI-SDS showed an average decrease in EG1 by 0.08±0.03 (P < 0.001) and EG2 by 0.07±0.03 (P = 0.012).
	-Participants in EG1 and EG2 attended weekly group meetings for 12 weeks. Each meeting lasts 60 minutes and involves 12-15 participants. When dietician and the psychologist co-conduct the meeting, they concentrate on cognitive behavioral modifications in the family lifestyle.	-There was no difference in BMI-SDS between the groups after 3 months (P = 0.440).
		-After 2 years of follow-up, BMI-SDS in EG2 reduced by 9% (P = 0.006) compared to the baseline.
	-CG treated with routine clinical follow-up.	-There was no difference in BMI-SDS across groups after the follow-up (P = 0.208).
	Education materials:	-The EG2 had higher attendance at meetings, indicating a significant correlation in the lowering of BMI-SDS after three months (r = 0.382, P = 0.005).
	-Focus on increasing physical exercise time.	Metabolic syndrome (MS):
	-Eating according to the health pyramid, which includes drinking plenty of water, eating plenty of fruits and vegetables, avoiding sugary beverages, and limiting fast food intake.	- EG1 has decreased excess adiposity by 23.1% (P = 0.027).
		- In all groups, the prevalence of MS risk factors did not change significantly.
	-Controlling screen time during the day.	- The MS rate among children dropped by 4.5% at 3 months (P = 0.109).
		Lifestyle changes and physical activity behaviors:
		- EG2 shows a decrease in fast-food consumption at the end of 3 months (P < .001) and a follow-up
		- CG had increased screen time by an average of 25 minutes per day at the end of the follow-up period (P = 0.032).
		- All groups had an increase in median (IQR) time of physical activity at the end of 3 months (30 minutes per week, P = 0.045).
Habib-Mourad (2014) [18] Lebanon	Educational program	Anthropometrics: NS change in WC in both EG& CG (p > 0.05). As well, BMI did not change between groups post intervention.
	‘Health-E-PALS’: "a program to promote Healthy Eating and Physical Activity in Lebanese school children". Based on SCT, this program addresses changing the behavior of students and changing the school environment (whole school approach).	Diet: Significant increase/decrease in odds ratio post intervention in EG compared to CG.
		- Eating a daily breakfast is 3.5 times greater in EG than in CG
		- Eating while watching TV is 56% less likely.
		- The odds of eating chips as snacks decreased by 86% and soft drinks by 69%.
		- The odds of purchasing chips from the school shop decreased by 84%, soft drinks by 88% and chocolate by 71%.
	It consists of three components:	Physical activity: Students in the EG were 40% more likely to play at recess than students in CG
	1) 12 interactive sessions once/week delivered by the researcher about various topics related to nutrition, physical activity and sedentary behaviours. CG: Students receive the usual curriculum during the same period.	Sedentary behaviours: NS difference in TV time spent and electronic games between groups (p > 0.05)
	2) A family program including meetings, health fairs, and recipes was sent home along with food samples.	Other: Significant increase (p < 0.001) for knowledge score by 2.8 points and self-efficacy by 1.7 points among EG only.
	3) Focus on school shops and family lunch boxes to make healthier food	

Changes in the school environment

Healthy Diets

In this review, several studies have implemented strategies for promoting healthy eating habits in schools [12,13,15-18]. These strategies include incorporating nutrition education into the curriculum, implementing interactive lessons for educating children and their parents about healthy eating habits, and reading food labels. Supporting healthy school breakfasts and lunches in accordance with dietary guidelines. These strategies align with other school-based intervention studies [19,20]. Highlighting the effectiveness of cooking workshops, studies showed these workshops build practical skills and positive associations with healthy food and encourage families to bring healthy lunches. By actively participating in meal preparation and cooking demonstrations, students learn food preparation techniques, ingredient selection, and portion control. This empowers them to make healthier choices in their daily lives. Hands-on workshops targeting parents and students in preparing nutritious snacks and meals for lunch box preparation and organizing a workshop to transform traditional food into healthy, delicious foods. Further, providing a booklet with recipes containing a variety of healthy breakfast foods has been shown to be effective [9,16,18]. These findings match those observed in earlier studies [21,22].

The school should adopt policies enforcing school environment and neighborhood shops to encourage healthy food choices and limit unhealthy snacks and beverages. In collaboration with stakeholders such as district supermarkets, school canteens, and cafeterias providers to offer a greater variety of healthy foods. As the following studies in this review suggested [12,16,18] In accordance with other studies done in the United States which have demonstrated a significant decrease in BMI by implementing a model in the District of Columbia and California according to the guidelines of the United States Preventive Services Task Force by implementing school wellness policies and addressing community partnerships [23].

Physical Activity and Decreasing Sedentary Time

Studies in this review focused on educating students about how to reduce their sedentary habits [12,14,15,17]. These include watching TV, spending a lot of time on social media, playing video games, and using a computer, laptop, or tablet. Increasing their sports time by offering fun and engaging active play programs, extending physical activity classes or incorporating short activity breaks throughout the day to increase overall activity levels and restrict time for exercise sessions incorporated into the school curriculum and involving parents in the intervention via assignments. The use of technology such as actigraphy [9] or pedometers [14] to track children's physical activity records including steps count, calories burned, sleep patterns, and hours has proven to be more effective in reducing children's weight. Moreover, incentives or reward systems are crucial to motivate children to follow healthy eating or physical activity advice [14,16]. It is important to note that all these strategies are aligned with other studies and systematic reviews from around the world [21,24,25]. 

Outcome components

A number of outcomes were studied, including weight-related measures, dietary behavior, physical activity, and sedentary behavior. Weight-related outcomes were reported in all studies as a change in BMI, a BMI-for-age Z-score (BAZ), or a BMI-standard deviation score (BMI-SDS). One study found a significant decrease in BAZ score (P = .003) [12], while two studies reported no significant difference in BAZ [15,16]. Only one study using BMI-SD conducted by Israel found the most substantial reduction in BMI-SD reduced by 9% (p = .006) after two years of follow-up was achieved by the intervention group which included children and their parents. Four studies revealed no significant change or difference in BMI [9,13,14,18]. Other anthropometric parameters include hip, waist, and neck circumferences; muscle mass, fat mass, fat-free mass; and triceps skin folds. Choudhury et al. [16] reported a significant increase in FM (p = 0.003), MM (p = 0.001), FFM (p = 0.001) in addition to HC and NC (p < 0.001), while Amini et al. [12] reported a significant decrease in HC (p < .001). 

Regarding lifestyle modification, it was noted that the programs had the greatest impact on weight loss, dietary habits, and sedentary time. The programs follow a whole-school approach by including children and their parents. In addition, they modify the school environment since a child spends half of his day at school. One such successful program is "Health-E-PALS”: "a program to promote Healthy Eating and Physical Activity in Lebanese school children" through interactive learning. By participating in this program, students gained a greater understanding of nutrition, increased their self-efficacy, and reduced their consumption of high-energy snacks and beverages [18]. In addition, Yackobovitch-Gavanet et al. [17] did weekly meetings with the parents for three months with a psychologist and nutritionist to modify the cognitive behaviours of the lifestyle of the family. This had a significant reduction in the children's BMI in the short and long term. It was found that the method of delivery and the number of sessions conducted had a considerable impact on lifestyle modification and behavior change. Studies that used online educational sessions or long distances between sessions (e.g., every month) [13], were less effective than those that utilized direct discussions or meetings with parents face-to-face [17,18].

Adaptability and recommendation for Saudi Arabia

A significant reduction or change in weight-related parameters was not expected, given that the studies included in this review lasted for an average of seven months. In contrast, lifestyle modification requires consistent follow-up over a longer period of time to achieve substantial results. A systematic review showed that the majority of studies targeting six- to 12-year-old children that proved effective on some indicators of adiposity involved long-term intervention periods >12 months [26]. In Eastern Mediterranean culture, being chubby as a child is a reflection of a healthy child from most parents' perspectives. Boodai et al. [15] found a high retention rate in family adherence to the education program. This may be related to parents' views since they didn't classify their children as obese or having a health condition that needed to be managed. Hence, we recommend incorporating a laboratory profile such as lipid, glucose, and metabolic syndrome for risk factors related to obesity. This will encourage parents to understand obesity's consequences objectively. Teachers are role models for their students, so it is essential to concentrate on teachers by providing them with knowledge and building their skills through a nutritious diet and physical activity. This is done by assigning them to workshops and courses so they can effectively teach and promote healthy habits in the classroom. In addition to empowering children to be ambassadors for healthy habits in their families and communities. Taking into consideration urbanization and high income, like in the Gulf Cooperation Council (GCC), along with modern house designs, has resulted in more sedentary lifestyle behaviors and fewer play activities for children. A number of solutions have been recommended, including providing active session breaks during the school day, which showed an effective strategy for reducing sedentary time and increasing physical activity time in accordance with a study done on students in Italy [27]. The interaction with the environment involves designing schools that include a running track, swimming pool, and backyard. This is to allow students to practice various exercises. Having a well-structured physical activity program that is unified for all students based on age groups and gender. Building special programs for students with chronic disease conditions like diabetes type 1 or students with special needs. To ensure the success and effectiveness of such programs, we must focus on the content, quality, duration, and priority of the physical activity intervention, along with teacher competence, as concluded by a systemic review conducted to promote physical activity in a school-based setting [25]. For long-term motivation, reward systems that encourage healthy eating, physical activity, and reducing sitting time are fundamental. For example, providing certificates or badges for students who achieve certain milestones. Students who follow healthy lifestyle habits may even receive incentives such as sports equipment or gift cards. Such as bringing a healthy, balanced breakfast and lunch box to school, participating in sports competitions, or completing the number of days and hours of physical activity in accordance with the recommended guidelines. In order to monitor and develop personalized plans for managing overweight and obese children, a team of primary care physicians, school nurses, and dietitians should be assembled. These plans include frequent check-ups, dietary counseling, physical activity recommendations, and ongoing support and education for both the child and their parents. Regular communication and collaboration between the healthcare team, the child's school, and the child's family are necessary for the successful monitoring and management of obesity and related risk factors. Implementing an effective reporting system between the Ministry of Education and the Ministry of Health will help close the loop efficiently. However, it can be challenging due to potential issues such as data privacy concerns, coordination between different government departments, and the need for standardized reporting formats. It will require close collaboration, clear communication channels, and a robust infrastructure to ensure relevant information is shared in a timely and secure manner. This will enable effective monitoring and management of obesity in children.

We recommend future studies on the current topic consist of well-designed interventional studies with a mixed-methods study design, a longer study duration, and a larger sample size. Develop an interventional program based on social cognitive theory, the social-ecological model, and ecological system theory. Since those theoretical frameworks showed the effectiveness of behavioral changes among schoolchildren to reduce obesity. This was according to a systematic review of GCC countries and a quasi-experimental study in Thailand [28,29].

## Conclusions

To maintain effective lifestyle and behavioral changes, there must be a clear set of objectives and a plan to follow to prevent relapse. School-based lifestyle programs can lower childhood obesity by involving all relevant stakeholders, such as teachers, parents, students, and schools, and using reliable instruments to track results to establish a healthy community. A program focusing only on parent education and materials without promoting relevant policy and environmental changes was found to be less effective. The use of incentives for children and family motivation is effective long-term. In order to confirm these findings, more research is required for a longer period of time, more than six months.
